# Plasmatic Levels of N-terminal Pro-BNP in Elderly Patients with Atrial Fibrillation and Heart Failure with Preserved Ejection Fraction

**Published:** 2017-04

**Authors:** Vasile Mircea ZĂGREANU, Dana POP, Mihnea ZDRENGHEA, Adela SITAR-TAUT, Bogdan CALOIAN, Dumitru Tudor ZDRENGHEA

**Affiliations:** 1. Emergency County Hospital, Baia Mare, Romania; 2. University of Medicine and Pharmacy, Cluj-Napoca, Romania

## Dear Editor-in-Chief

Heart failure with preserved ejection fraction has become an epidemiological problem in the last years. Mortality is similar as in patients with heart failure and reduced left ventricular ejection fraction (1). The presence of atrial fibrillation and heart failure worsens the prognosis of patients. Cardiac peptides are significant markers for the development of both conditions. We investigated the impact of atrial fibrillation on serum levels of NT-pro-BNP in elderly heart failure patients as compared to sinus rhythm patients.

The study included 101 heart failure patients with preserved ejection fraction (>50%) selected from the databases of the general practitioners in Baia-Mare and Cluj-Napoca in 2015.

The selected patients were informed about the study protocol and signed informed consent was taken from them. The study was carried out in agreement with The Code of Ethics of the World Medical Association (Declaration of Helsinki) for experiments involving humans.

The patients were divided into two groups: group 1: 49 patients with atrial fibrillation (72.12±6.78 yr, 42.9% women) and group 2: 52 patients in sinus rhythm (71.68±7.69 yr, 71.2% women).

**Fig. 1:**
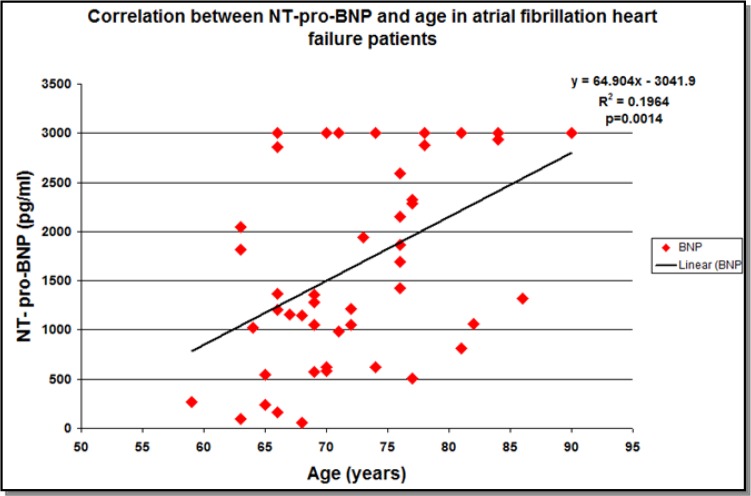
Correlation between NT-pro-BNP and age in atrial fibrillation heart failure patients

When taking into account all heart failure patients, the mean NT-pro-BNP value was high: 1229.44 ± 1040.1178 pg/ml. On the contrary, mean plasmatic value of NT-pro-BNP was 843.40±938.05 pg/ml in sinus rhythm patients (group 2), significantly lower than NT-pro-BNP levels in heart failure and atrial fibrillation patients (group 1)- 1639.12±993.03 pg/ml, (*P*=0.0001). There was a direct correlation between NT-pro-BNP level and age: all patients-correlation coefficient r=0.1977 (95% CI-0.0136 to 0.3794), *P*=0.048; group 1 correlation coefficient r=0.4432 *P*=0.0014 (95% CI-0.1851 to 0.6441) (Fig. 1); but not in group 2 correlation coefficient r=−0.01 (95% CI −0.2863 to 0.264), *P*=NS. Atrial fibrillation is associated with natriuretic hormones increase even in the absence of heart failure (2). The mean values of NT-pro-BNP in atrial fibrillation ranged between 800 and 1100 pg/ml and they were not associated with the duration of atrial fibrillation or with the size of the atrium (3).

In our study, there was a direct correlation between NT-pro-BNP level and age: all patients-correlation coefficient r=0.1977 (95% CI-0.0136 to 0.3794), *P*=0.048; group 1 correlation coefficient r=0.4432 *P*=0.0014 (95% CI-0.1851 to 0.6441)-Fig. 1; but not in group 2- correlation coefficient r=−0.01 (95% CI −0.2863 to 0.264), *P*=NS. NT-pro-BNP values increase with age, even in the absence of cardiovascular pathology in elderly patient. In effect, every 100 pg/ml increase in the NT-pro-BNP levels is associated with a 35% increase in the relative risk of death (4). In the Multi-Ethnic Study of Atherosclerosis, NT-pro-BNP was an important predictor for atrial fibrillation (5).

There was also a significant correlation between NT-pro-BNP and LV ejection fraction in all heart failure patients (correlation coefficient r=−0.3168, *P*=0.0012, 95% CI −0.4823 to −0.1293). When analyzing the two groups apart, only sinus rhythm patients displayed a significant indirect correlation between NT-pro-BNP levels and LV ejection fraction (correlation coefficient = −0.198, *P*=0.16 in atrial fibrillation patients vs correlation coefficient =−0.435 *P*=0.0019). The respective peptide is a major predictor for the evolution of heart failure (1). In neither groups, there was a significant correlation between left atrium size and NT-pro-BNP values (*P*=0.247, respectively *P*=0.561). At the same time, NYHA class was directly correlated with NT-pro-BNP values in all patients, in group 2, but not in group 1. On the contrary, in a study including 32 patients with atrial fibrillation, there was a significant correlation only between NT-pro-BNP values and left atrium size, and not between the latter and NANP levels (6). In fact, three factors contributed to the increase in NT-pro-BNP levels in the patients of cohort 1: old age, heart failure, even with preserved eject fraction, and atrial fibrillation. Elderly heart failure patients with preserved left ventricular ejection fraction and atrial fibrillation display increased levels of plasmatic NT-pro-BNP as compared to those with sinus rhythm, which proves that atrial fibrillation determines a more substantial increase in NT-pro-BNP levels, even in patients with heart failure.
